# Brain aging and neurodegeneration: from a mitochondrial point of view

**DOI:** 10.1111/jnc.14037

**Published:** 2017-05-14

**Authors:** Amandine Grimm, Anne Eckert

**Affiliations:** ^1^ University of Basel Transfaculty Research Platform Molecular & Cognitive Neuroscience Neurobiology Laboratory for Brain Aging and Mental Health Basel Switzerland; ^2^ University of Basel Psychiatric University Clinics Basel Switzerland

**Keywords:** aging, bioenergetics, brain, Mitochondria, mitochondrial dynamics

## Abstract

Aging is defined as a progressive time‐related accumulation of changes responsible for or at least involved in the increased susceptibility to disease and death. The brain seems to be particularly sensitive to the aging process since the appearance of neurodegenerative diseases, including Alzheimer's disease, is exponential with the increasing age. Mitochondria were placed at the center of the ‘free‐radical theory of aging’, because these paramount organelles are not only the main producers of energy in the cells, but also to main source of reactive oxygen species. Thus, in this review, we aim to look at brain aging processes from a mitochondrial point of view by asking: (i) What happens to brain mitochondrial bioenergetics and dynamics during aging? (ii) Why is the brain so sensitive to the age‐related mitochondrial impairments? (iii) Is there a sex difference in the age‐induced mitochondrial dysfunction? Understanding mitochondrial physiology in the context of brain aging may help identify therapeutic targets against neurodegeneration.

This article is part of a series “Beyond Amyloid”.

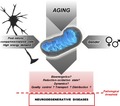

**Abbreviations used**
AD: Alzheimer's disease
COX: cytochrome c oxidase
Drp1: dynamin‐related protein 1
ER: estrogen receptor
ETC: electron transport chain
Fis1: fission protein 1
GSH/GSSG: reduced/oxidized glutathione
Mfn1/2: mitofusin ½
mPTP: mitochondrial permeability transition pore
mtDNA: mitochondrial DNA
NRF‐1: nuclear respiratory factor 1
Opa1: optic atrophy 1
OXPHOS: oxidative phosphorylation
PDH: pyruvate dehydrogenase
PGC‐1α: peroxisome proliferator‐activated receptor gamma coactivator‐1α
PINK1: PTEN‐induced putative kinase 1
ROS: reactive oxygen species
SAM: senescence accelerated mice
SOD: superoxide dismutase
TFAM: mitochondrial transcription factor A

The aging process was defined by Harman as a ‘progressive accumulation of changes with time that are associated with or responsible for the ever‐increasing susceptibility to disease and death which accompanies advancing age’ (Harman 1981). Indeed, age is the main risk factor for prevalent diseases, including neurodegenerative disorders [reviewed in (Niccoli and Partridge 2012)]. With the aging of populations, the number of persons affected by brain disorders will increase, as for example for Alzheimer's disease (AD) which prevalence is expected to double within the next 20 years (Prince *et al*. 2013).

The brain is a high energy consuming organ that requires about 20% of body basal oxygen to fulfill its function. Thus, it is not surprising that disturbances in brain energy metabolisms lead to disease, ranging from subtle alterations in neuronal function to cell death and neurodegeneration. Cellular energy is mainly produced via oxidative phosphorylation (OXPHOS) taking place within mitochondria. These organelles are often compared to powerhouses, providing cellular energy under the form of ATP molecules. Mitochondria produce the energy required for almost all cellular processes, from cell survival and death, to the regulation of intracellular calcium homeostasis, synaptic plasticity and neurotransmitter synthesis (Mattson *et al*. 2008). However, when mitochondria fulfill their physiological functions, they can also be compared to a double‐edged sword that, on one hand, produces the energy necessary for cell survival, and on the other hand, induces the formation of reactive oxygen species (ROS) that can be harmful for cells when produced in excess with mitochondria as the first target of toxicity (see section: *‘* Mitochondria and the free radicals theory of aging’).

Given the central role of mitochondria in energy metabolism as well as in the regulation of reduction‐oxidation (redox) homeostasis, the study of age‐related mitochondrial impairments is becoming of growing interest in order to unravel the mechanism leading to either normal aging or to neurodegenerative disorders. Thus, in this review, we aim to give insights about the role of mitochondria in the aging process, in particular, what may make the brain so sensitive to mitochondrial impairments? We aim to reflect about the role of mitochondria in the aging process, with a specific focus on the brain. Starting from the ‘mitochondrial theory of aging’, we will discuss why the brain seems to be so sensitive to the age‐related mitochondrial impairments. Finally, we will give insights about sex differences linked to mitochondrial function in aging.

## Mitochondria and the free radicals theory of aging

The ‘free‐radicals theory of aging’ was stated for the first time by Harman in 1956 (Harman 1956), and postulates that aging, as well as age‐associated degenerative diseases, is a consequence of free radicals attacks on cells and tissues. But how these ‘harmful’ molecules are they generated?

**Figure 1:**
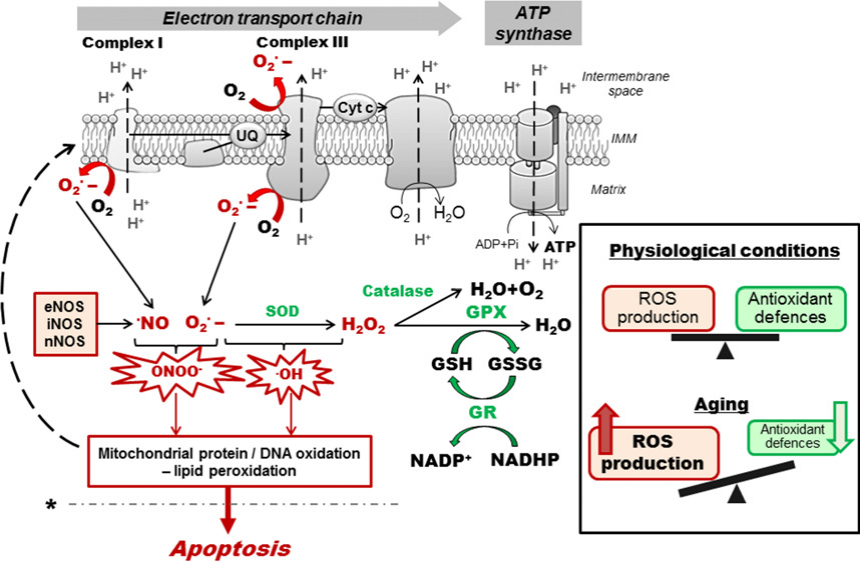
Reactive oxygen species formation and detoxification. Superoxide anion radicals (O_2_˙‐) are mainly generated by the mitochondrial complexes I and III during electron transfer through the electron transport chain (ETC.). O_2_˙‐ can interact with •NO, produced by nitric oxide synthase (NOS), to generate peroxynitrite (ONOO‐) or can also react to form •OH (hydroxyl radical). Detoxification involves the enzymatic activity of superoxide dismutase (SOD) that converts O_2_˙‐ to H_2_O_2_ and may diffuse to the cytoplasmic compartment where glutathione peroxidase (GPX) and catalase convert H_2_O_2_ to H_2_O. In physiological conditions, reactive oxygen species (ROS) production and detoxification are in balance. In aging, increase in ROS production and/or defects in the antioxidant system may induce oxidative stress leading to protein and DNA oxidation as well as lipid peroxidation, which can in turn affect the ETC. and exacerbate ROS production (dashed arrow). This may trigger a vicious cycle of oxidative stress that lead to cell death by apoptosis when a pathological threshold is passed (dashed line marked by *). eNOS/iNOS/nNOS; endothelial/inducible/neuronal nitric oxide synthase, GSH/GSSG; reduced/oxidized glutathione, GPX; glutathione peroxidase, GR; glutathione reductase, IMM; inner mitochondrial membrane.

ROS derive mainly from the OXPHOS taking place within mitochondria. Indeed, the production of ATP by mitochondria requires about 85% of oxygen (O_2_) consumed by cells. Mitochondrial complex IV reduces O_2_ into H_2_O using electrons derived from NADH or FADH_2_ driven in the respiratory chain. An inevitable byproduct of electron transport chain (ETC) activity is the formation of superoxide anion radicals (O_2_˙^−^), mostly by complexes I and III (Jezek and Hlavata 2005) (Fig. 1).

In physiological conditions, ROS are involved in processes such as immune response, inflammation, as well as synaptic plasticity, learning and memory (Kishida and Klann 2007; Veal *et al*. 2007). However, when produced in excess, those molecules can induce oxidative stress, damaging proteins and DNA, and inducing lipid peroxidation, with the corresponding mitochondrial structures as the first targets of toxicity. ONOO^−^ can induce nitration of proteins and impair their function (Goldstein and Merenyi 2008), and •NO has been shown to inhibit the mitochondrial complex IV activity by competitive binding on its oxygen site (Brown and Borutaite 2002). Furthermore, since mitochondrial DNA (mtDNA) is localized close to free radical production sites, it is directly in contact with those molecules and can exhibit oxidative damages. Oxidative stress can trigger cell death and has been implicated in the pathogenesis of many neurodegenerative diseases, such as Alzheimer's disease (Keil *et al*. 2004; Leuner *et al*. 2012).

To counteract these oxidative attacks, cells are endowed with robust antioxidant defenses, involving superoxide dismutase (SOD) and the glutathione (GSH) system (Fig. 1). However, when an imbalance between free radical production and detoxification occurs, ROS production may overwhelm antioxidant defenses, leading to impairments of cellular function. This phenomenon is observed in many pathological cases involving mitochondrial dysfunction, as well as in aging (Grimm *et al*. 2016b).

## Redox homeostasis and mitochondrial bioenergetics in brain aging

A growing body of evidence highlights bioenergetic impairments as well as disturbances in the reduction‐oxidation (redox) homeostasis in the brain with increasing age.

In human, postmortem investigation of different brain regions revealed a progressive age‐related raise in protein nitration and oxidation, together with a decrease in SOD, catalase and GSH reductase activity, as well as complex I activity, mainly in the hippocampus and frontal cortex (postmortem brain samples from individuals between 0.01 and 80 years old) (Venkateshappa *et al*. 2012). In line, *in vivo* monitoring of GSH content in the human brain from healthy subjects showed a gradual decrease in this antioxidant enzyme in old (± 56 years) compared to young (± 26 years) individuals (Mandal *et al*. 2012). Interestingly, Peskind and co‐workers showed a 10% increase in free radical injury in the brain of medically healthy and cognitively normal adults from age 45 to 71 years, using cerebrospinal fluid F2‐isoprostane as biomarker (Peskind *et al*. 2014). Besides, this age‐related increase in brain oxidative stress was even greater in individuals with high body mass index and in smokers, highlighting the influence of lifestyle in the processes of aging.

Similar data were obtained in different animal models. In a recent study performed in a nonhuman primate model (Rhesus monkeys Macaca mulatta), Pandya and colleagues provided evidence of a decreased mitochondrial bioenergetics (ATP synthesis capacity and pyruvate dehydrogenase activity) and calcium buffering in the putamen of old animals (20–25 years old) compared with young animals (6–8 years old) (Pandya *et al*. 2015). These age‐related mitochondrial impairments in the basal ganglia were correlated with reduced locomotor activity and movement speed in older animals.

In rats, an increase in oxidative DNA damages and peroxide generation, parallel to a decrease in mitochondrial membrane potential was observed in the brain and liver of old (27 months) when compared to young (4 months) animals (Sastre *et al*. 1998). Interestingly, a treatment with Ginkgo biloba extract (EGb 761), known to have antioxidant properties, partially prevented these alterations, highlighting the role of oxidative stress in age‐related mitochondrial impairments (Sastre *et al*. 1998). Similarly, the analyses of 24‐month‐old rat brains revealed a decrease in manganese SOD (MnSOD) activity when compared to brains from 7‐month‐ old animals (Navarro and Boveris 2004). Again, this decrease was paralleled to a loss of mitochondrial complex I and IV activity.

In line with these findings, a shift to a pro‐oxidized state was measured in the brain of old (21 months) compared with young (3 months old) mice, associated with a decrease in the GSH/GSSG ratio (reduced/oxidized glutathione) (Rebrin *et al*. 2007). Remarkably, these alterations were mainly found in the cortex, hippocampus and striatum that are brain regions linked to age‐related loss of cognitive function. Functional mitochondrial impairments were also evident in the brain of 24‐month‐old NMRI (Swiss‐type) mice compared to 4 months old ones, with deficits in the activity of complex I, basal mitochondrial membrane potential as well as mitochondrial respiratory control ratio (RCR, indicator of mitochondrial coupling) (Leuner *et al*. 2007). Of note, the decline in the RCR was particularly evident in the brain of female mice during reproductive senescence (Yao *et al*. 2010; Klosinski *et al*. 2015) (see section: ‘Sex differences in age‐induced mitochondrial dysfunction*’*). The opening of the mitochondrial permeability transition pore (mPTP), resulting in membrane depolarization and uncoupling of the OXPHOS, was also involved in the aging process (Toman and Fiskum 2011). In a recent study comparing parameters of mPTP in the brain, the authors highlighted a decreased threshold Ca^2+^ concentration to induce mPTP opening in mitochondria isolated from old (18 months) compared to young (3 months) rats (Krestinina *et al*. 2015).

In order to study in more detail the mechanisms underlying age‐related mitochondrial impairments, animal models were developed, including the senescence accelerated mice (SAM) strains. For instance, in SAMP8 mice (accelerated senescence‐prone 8), a naturally occurring mouse line that displays a phenotype of accelerated aging, impairments in the brain antioxidant system were observed, such as a reduction in GSH level, in SOD activity (MnSOD and Cu/Zn‐SOD) as well as catalase activity, compared to age‐matched accelerated senescence‐resistant 1 (SAMR1) mice (Kurokawa *et al*. 2001; Shi *et al*. 2010). These failures of antioxidant defenses were again coupled with disturbances of the OXPHOS, translated by a decrease in complex I, complex IV and ATP synthase activity, leading to a reduction in ATP production (Xu *et al*. 2007; Shi *et al*. 2010). More recently, Wang and collaborators showed similar results in SAMP10 mice (a substrain of the SAM) (Wang *et al*. 2015). SOD activity was decreased already in 8‐month‐ old mice, together with an increase in lipid peroxidation, which may contribute to the appearance of age‐related impairments in learning and memory.

Consistent with these findings, Nakahara and collaborators provided evidence of an age‐related decrease in the RCR of mitochondria isolated from the liver of SAMP8 mice when compared to SAMR1 mice at the same age (Nakahara *et al*. 1998). Indeed, in SAMP8 mice, RCR tended to decline at 12 months of age, and dramatically decreased at 18 months of age. OXPHOS alterations were also observed in the heart of 12‐month‐ old SAMP8 mice when compared to age‐matched SAMR1 animals (Nakahara *et al*. 1998). The authors suggested that at 18 months of age, the respiratory control values may be insufficient to maintain ATP levels necessary for keeping normal cell metabolism. In addition, Omata and collaborators showed age‐related changes in cerebral energy production in the 2‐month‐ old SAMP8 followed by a decrease in mitochondrial function compared with SAMR1 (Omata *et al*. 2001).

Taken together, these findings emphasize that brain aging is marked by (i) a decrease in antioxidant defenses, (ii) an increased oxidative stress, and (iii) deficits in the mitochondrial OXPHOS system. Thus, we can speculate that the enhancement of ROS production may trigger a ‘vicious cycle’ of oxidative stress, leading to mitochondrial dysfunction and apoptosis. But one can ask the question whether increased ROS levels is a primary consequence of mitochondrial dysfunction or whether a primary defect in the free radical scavenging activity is responsible for an abnormal respiratory function. Besides, as mentioned above, mitochondria are not only the major producers of ROS, but they are also the main targets of ROS toxicity. Thus, the antioxidant system should be very efficient to prevent oxidative damages, especially in the paramount organ that is the brain.

## Neuronal mitochondria: what makes our brain so special?

The brain is a remarkable organ composed by highly differentiated cells that populate different anatomical regions. Neurons are polarized cells with different morphology, according to their role and their localization. These post‐mitotic and excitable cells have really high energy requirements: (i) to maintain their membrane potential allowing the propagation of electric signals, (ii) to re‐establish the ion balance after the firing of action potential (e.g. via the Na^+^/K^+^ ATPase activity), (iii) to trigger the release of neurotransmitters by fusion of vesicles to the plasma membrane, (iv) to allow the recapture of neurotransmitters from the synaptic cleft. Glucose oxidation is the most relevant source of energy in the brain, since other fuel sources, such as fatty acid oxidation, have an ATP generation rate too slow to sustain neuronal energy demands, and produce too much ROS that may cause oxidative stress [reviewed in (Schonfeld and Reiser 2013)]. In consequence, neurons rely almost exclusively on the mitochondrial OXPHOS system to fulfill their energy needs supplied under the form of ATP.

Some studies aimed to compare mitochondrial properties in different organs, including the brain. For instance, mitochondria isolated from rat liver, kidney, brain and skeletal muscle showed significant and similar proton leak, but the phosphorylating systems appeared to be more active in the brain and the muscle (Rolfe *et al*. 1994). Differences in the rat mitochondrial proteome were also observed when comparing the kidney, liver, heart, skeletal muscle, and brain (Reifschneider *et al*. 2006). Moreover, each organ possesses different protein composition, especially in the expression of proteins involved in the OXPHOS system. Interestingly, ROS derived from mitochondria appear to be generated by distinct complexes according to the organ considered. Indeed, the Barja group reported that oxygen radicals are exclusively generated by the mitochondrial complex I in the brain (non‐synaptic mitochondria), whereas they can be produced by both complex I and III in the heart (Barja 1999). Surprisingly, no significant variations in free radical production were found when comparing young with old rodents. However, it appeared that animals (mammals and birds) with higher longevity displayed a lower rate of ROS production in the heart, brain, lung, kidney, and liver compared to short living ones, which is in agreement with the ‘rate of living theory’ (inverse relationship between longevity and metabolic rate) [reviewed in (Barja 1999)].

It is important to note that neurons are post‐mitotic cells with a life span similar to that of the whole organism (Terman *et al*. 2010). Unlike in other organs, such as the skin or the liver, damaged neurons are not (or rarely) replaced during life, stressing the importance of protecting systems, including antioxidant defenses, to maintain neuronal integrity and survival. Post‐mitotic cells, such as neurons, seem to be more sensitive to the accumulation of oxidative damages compared to dividing cells, and are more prone to accumulating defective mitochondria during aging (Kowald and Kirkwood 2000; Terman *et al*. 2010).

In addition, to bring a higher degree of complexity, neurons are exceedingly compartmentalized, comprising structures like: cell body, axon, dendrites, and even more specific compartments that are the synapses. Consequently, a proper mitochondrial distribution is paramount to sustaining the energy requirement at specific locations within the different neuronal compartments (Obashi and Okabe 2013; Lin and Sheng 2015; Pernas and Scorrano 2016). Thus, it is not so surprising that synaptic mitochondria, which need to sustain the energy required for synaptic activity, present functional differences when compared to non‐synaptic mitochondria. Indeed, peroxide production was found higher in synaptic mitochondria of rats, compared to non‐synaptic ones (Borras *et al*. 2003, 2010). Interestingly, aging seems to accentuate the differences between these two populations of mitochondria. In 14‐month‐ old rats, state 3 respiration was significantly decreased only in synaptic mitochondria, when compared with 3‐month‐ old rats (Lores‐Arnaiz and Bustamante 2011). Besides, a higher susceptibility to calcium insult was observed only in synaptic mitochondria of old animals. In non‐synaptic mitochondria, oxygen consumption was not significantly affected by aging, and both populations of mitochondria generated higher levels of peroxide in 14‐month‐ old animals compared to young animals. Similar data were obtained in mice where basal respiration as well as respiration driving proton leak were significantly decreased in synaptosomes from old mice (17 months) compared with young mice (3 months) (Lores‐Arnaiz *et al*. 2016). No differences were observed in non‐synaptic mitochondria. Again, only synaptosomal mitochondria were susceptible to undergoing calcium‐induced depolarization in old animals.

Taken together, these findings suggest that mitochondria located at the nerve terminals are more sensitive to age‐related dysfunction and oxidative damage compared with non‐synaptic mitochondria. Again, this emphasizes the importance of proper distribution of mitochondria to sustaining the spatial and temporal demand of energy in neurons, which differs within the axons and synapses compared to dendrites and cell body but also to maintaining synaptic plasticity in the adult brain (Amadoro *et al*. 2014; Lin and Sheng 2015; Todorova and Blokland 2016). Thus, the accumulation of mitochondrial dysfunctions (such as mtDNA mutations, increased oxidative stress) coupled with defects in mitochondrial transport/distribution may play a prominent role in the appearance of age‐related diseases with synaptic/neuronal degeneration. To preserve the cells against this scenario, mitochondria possess an endogenous quality control allowing the mixing of mitochondrial content (mitochondrial dynamics), as well as the degradation of defective organelles (mitophagy).

## Age‐related mitochondrial defects and the importance of mitochondrial dynamics in aging

### Mitochondrial fusion/fission and mitophagy

Mitochondria possess a residual genome (approximately 16 kilobase) coding for 13 proteins essential for mitochondrial respiratory chain function, which make them unique organelles carrying autonomous DNA (Friedman and Nunnari 2014). It appears that the quality control of mtDNA replication is not as efficient as nuclear DNA (nDNA), resulting in an increased risk of mtDNA mutations (DeBalsi *et al*. 2016). Fortunately, to avoid the accumulation of such mutations, mitochondria are remarkably dynamic organelles that divide and fuse in order to maintain a homogenous mitochondrial population by content mixing (mtDNA, metabolites, and proteins), quality control and distribution of mitochondria within the cell (Chan 2012).

**Figure 2:**
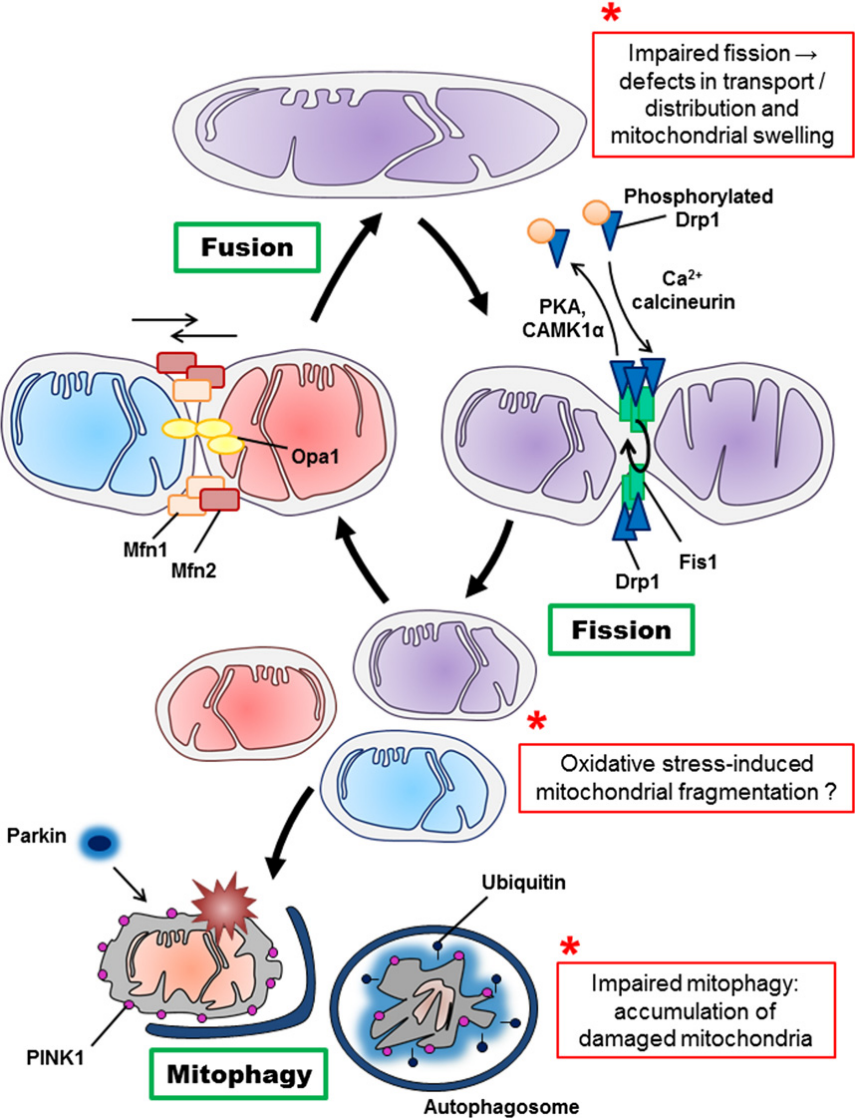
Schematic mechanisms of mitochondrial fusion, fission and mitophagy. Mitochondria cyclically shift between elongated (tubular) and fragmented state. The localization, as well as some interactions and modifications of the principal proteins involved in the two processes are shown. Once dephosphorylated, DRP1 (dynamin‐related protein 1) is recruited to the outer membrane by FIS1 (fission protein 1). The oligomerization of DRP1 is followed by constriction of the membrane and mitochondrial fission. Following the fission event, the mitochondrion can either be transported, or enter in fusion again. The pro‐fusion proteins mitofusin 1 and 2 (MFN1/2) on the outer membrane and optic atrophy 1 (OPA1) on the inner membrane) oligomerize to induce fusion of the membranes. Defective mitochondrion accumulates PINK1 kinase (PTEN‐induced putative kinase 1), recruiting the E3 ubiquitin ligase parkin, which ubiquitylates mitochondrial proteins and triggers mitophagy. The potential effects of aging on mitochondrial dynamics are marked by *. CAMK1α; Ca^2+^/calmodulin‐dependent protein kinase Iα, PKA: protein kinase A

Mitochondrial fission is one of two opposing mechanisms regulating mitochondrial dynamics. This process enables the renewal, redistribution and proliferation of mitochondria. It involves evolutionary conserved dynamin‐related GTPases, such as the dynamin‐related protein 1 (Drp1), master mediator of the fission, as well as fission protein 1 (Fig. 2) (Pernas and Scorrano 2016). The activity of DRP1 is highly regulated by post‐translational modifications, namely by phosphorylation/dephosphorylation involving enzymes such as for example the calcium/calmodulin‐dependent protein kinase 1α, protein kinase A or Ca^2+^ calcineurin (Campello and Scorrano 2010). The inactive form of DRP1 is dispersed in the cytosol and the activation by dephosphorylation is required to target mitochondrial membrane. Deletions or mutations in genes coding for Drp1 and fission protein 1 result in aberrant mitochondria morphology (hyperfused network), heterogeneous population of mitochondria with non‐uniform mtDNA distribution, varied ability to produce ATP, increased capacity to generate reactive oxygen species and increased susceptibility of cells to undergoing apoptosis (Knott *et al*. 2008; Oettinghaus *et al*. 2016).

The mechanism counteracting the fission is the mitochondrial fusion. This allows mitochondria to interact and communicate with each other, and facilitates mitochondrial movement and distribution across long distances (Chan 2006). Fusion events are particularly important for the enrichment of mtDNA via dilution of mutations. This two‐step process also requires the action of two evolutionarily distinct dynamin‐related GTPases (Fig. 2). The fusion of mitochondrial outer membranes is controlled by mitofusin 1 and 2 (Mfn1/2), whereas optic atrophy 1 (Opa1) controls inner membrane fusion (Campello and Scorrano 2010; Friedman and Nunnari 2014). Mitochondrial fusion is essential to maintaining a homogeneous mitochondrial population and ensures inter‐complementation of mtDNA (Chan 2006). Mutations in Mfn2 or Opa1 cause the autosomal dominant peripheral neuropathy, Charcot Marie‐Tooth disease 2A, and autosomal dominant optic atrophy respectively, and result in an extensive fragmentation of the mitochondrial network (Knott *et al*. 2008; Carelli *et al*. 2015).

Fusion/fission activity is also integrated with mitochondrial quality control pathways allowing the detection and removal of aged or damaged mitochondria through a specific form of autophagy, termed mitophagy (Fig. 2) (Twig and Shirihai 2011; Youle and Narendra 2011; Haroon and Vermulst 2016). The exact mechanism underlying mitophagy, more specifically what triggers mitophagy, remains to be elucidated in more detail. It has been proposed that damaged mitochondria present a decreased mitochondrial membrane potential [reviewed in (Twig and Shirihai 2011)]. Uncoupled mitochondria accumulates the protein PTEN‐induced putative kinase 1 at the surface of the mitochondrial outer membrane (Twig *et al*. 2008; Youle and Narendra 2011) recruiting the E3 ubiquitin ligase parkin specifically to the damaged mitochondrion (Fig. 2). Then, parkin ubiquitylates mitochondrial proteins leading to the formation of an autophagosomes and the digestion of the mitochondrion. This process mediates mitochondrial quality control.

In summary, when mitochondria fuse, they mix their membranes, matrix and inter‐membrane space, including all their content (lipids, proteins, metabolites and mtDNA) (Fig. 2). After this mixing, mitochondria can divide, sharing equally their new content between two daughter organelles. When a damaged mitochondrion is detected, it is eliminated from the fusion/fission cycle by mitophagy, guaranteeing a homogenous and healthy mitochondrial population. Thus, it is not surprising that defects in mitochondrial dynamics and mitochondrial quality control system may lead to cellular impairments, and was proposed to be involved in the process of aging and neurodegeneration (Knott *et al*. 2008; Chauhan *et al*. 2014).

### Implication of mitochondrial dynamics in aging

DeBalsi and collaborators recently highlighted in a review that defects in the mtDNA replication machinery result in the accumulation of mutations, leading to decreased ETC. activity and impaired mitochondrial respiration (DeBalsi *et al*. 2016). In addition, mounting evidence presents the accumulation of mtDNA mutations over time as a central mechanism driving to aging and age‐related diseases (Kujoth *et al*. 2005; Santos *et al*. 2013; Aon *et al*. 2016; DeBalsi *et al*. 2016; Kauppila *et al*. 2016; Scheibye‐Knudsen 2016). Of note, complex I is particularly susceptible to the aging process because the mtDNA encodes 7 of the 13 subunits building this mitochondrial complex (Paradies *et al*. 2010). Mutations in these genes often lead to diseases with varying phenotypes and severity depending on the mutational load (Nunnari and Suomalainen 2012).

In order to examine into more details the possible causative effects of the age‐related accumulation of mtDNA mutations, a mouse model was developed, expressing a proof‐reading‐deficient version of the nucleus‐encoded catalytic subunit of mtDNA polymerase (Zhang *et al*. 2000; Trifunovic *et al*. 2004). These ‘mtDNA mutator mice’ present significant increases (3–5 fold) in the levels of point mutations, as well as increased amounts of deleted mtDNA, associated with reduced lifespan and premature onset of ageing‐related phenotypes (weight loss, reduced subcutaneous fat, alopecia, osteoporosis, reduced fertility, and heart enlargement). The study of mitochondrial OXPHOS revealed severe respiratory chain dysfunction in embryonic fibroblasts from mtDNA mutator mice, but, interestingly, no increase in ROS production or oxidative damages in peripheral tissues (liver and heart) (Trifunovic *et al*. 2005). Deeper investigations were performed to assess whether mtDNA mutations or deletions were the driving force of the progeroid phenotype, especially in the brain. For instance, the analyses of the brain mitochondrial transcriptome and proteome of mtDNA mutator mice revealed that mitochondrial respiratory chain proteins (also those encoded in the nucleus) were specifically decreased in abundance in the brains, but, surprisingly, no significant changes were observed in mRNA expression (Hauser *et al*. 2014). These data suggested that changes in the mitochondrial proteome occurred at the post‐translational level. Thus, the characterization of mutator mice provided a causative link between mtDNA mutation/deletion and ageing phenotypes in mammals, but the underlying mechanisms remain unclear, and further investigations need to be performed, especially to understand the role of the age‐related accumulation of mtDNA in brain aging.

Mitochondrial dynamics seem to be a protective factor safeguarding mitochondria integrity in the face of mtDNA mutations. Indeed, on one hand, it was shown in the skeletal muscle that the absence of fusion, as a result of the ablation of Mfn1 and Mfn2, leads to the accumulation of point mutations and deletions in the mitochondrial genome, coupled with severe mitochondrial dysfunction and muscle atrophy (Chen *et al*. 2010). On the other hand, absence of fusion, because of Drp1 ablation in neurons of the adult mouse forebrain, causes alterations in mitochondrial morphology and transport to the synapse, together with a decrease in oxygen consumption and ATP production (Oettinghaus *et al*. 2016). Moreover, the loss of Drp1 affected synaptic transmission and memory function, demonstrating the critical role of mitochondrial fusion/fission activity in brain function. In line with these recent findings, in postmitotic Purkinje cells, Drp1 deletion results in mitochondrial swelling, impaired ETC. activity (including complex I and IV activity), increased oxidative damages, ubiquitination of mitochondria, accumulation of autophagy markers, leading to neurodegeneration in the cerebellum (Kageyama *et al*. 2012). Interestingly, the treatment of Drp1 KO cells with antioxidants (N‐acetylcysteine and MitoQ) was able to reduce mitochondrial swelling and cell death. Altogether, these data suggest that mitochondrial fission plays a paramount role in neuronal survival by acting as a quality control mechanism, suppressing oxidative damages.

As mentioned above, the aging process is associated with an increase in brain oxidative stress that may induce an accumulation of mtDNA mutations. Thus, efficient mitochondrial dynamics appears to be crucial in order to maintain a healthy organelle population. And when this protective system is impaired during aging, this may lead to pathological conditions (Chauhan *et al*. 2014). Strikingly, only few studies were focused on mitochondrial fusion/fission activity in the brain during aging. Using a proteomic approach, Stauch and colleagues investigated the age‐related changes in the expression of proteins in mice synaptosomal mitochondria (Stauch *et al*. 2014). They showed alterations in the expression of fusion/fission proteins, namely an increase in Drp1 expression between 5 and 12 months of age, and a decrease from 12 to 24 months. In parallel, Mfn1/2 and Opa1 decreased from 5 to 12 months and increased from 12 to 24 months. Altogether, these data suggested that synaptosomal mitochondria were shifted to a pro‐fusion state in aged animals.

Interestingly, in a study investigating the longevity of a specific rat strain (long‐living Fischer 344 ×  Brown Norway strain), a doubling of Drp1 and Mfn2 protein level was observed in the liver of 32‐month‐ old animals (AL‐32) compared to 28 months old (AL‐28) (Picca *et al*. 2016). Moreover, a positive correlation between the fusion index and the mtDNA content was observed in some of these animals, suggesting that in aged animals: (i) the prevalence of fusion might ensure a stable mtDNA content and mitochondrial network, (ii) functional mitochondrial dynamics may explain the longevity of the AL‐32 rats. These data need to be confirmed with a higher animal number (in this pilot study, only five animals were investigated per group).

As part of the mitochondrial quality control system, mitophagy plays an important role in removing damaged mitochondria from the cells (Fig. 2). Diot and collaborators recently showed an age‐related decline in mitophagy in skin fibroblasts of healthy donors (0–81 years old) (Diot *et al*. 2015). Impaired mitophagy may contribute to the decline of mitochondria function leading to the aging phenotype (reviewed in (Diot *et al*. 2016)). Although mounting evidence highlighted the implication of mitophagy in neurodegenerative diseases, especially in familial forms of Parkinson's disease [reviewed in (Batlevi and La Spada 2011)], its exact role in the regulation of neural function and neurodegeneration remains unknown in the context of brain aging. In a study performed on Drosophila, ubiquitous or neuron‐specific up‐regulation of the E3 ubiquitin ligase parkin (see (Fig. 2)) induced: (i) an increased lifespan in flies, (ii) reduced levels of protein aggregation during aging, and (iii) a modulated mitochondrial activity (citrate synthase and complex I activity) and dynamics (reduces mitofusin levels in young and aged flies) (Rana *et al*. 2013). Thus, these findings provided new insights into the mechanisms regulating mitophagy in brain aging and suggested that parkin protein may play a key role in this process. Of note, increased mitophagy was observed in skeletal muscle as well as in erythroid cells from mtDNA mutator mice, but this was not sufficient to protect mitochondria from the massive mtDNA damage (Joseph *et al*. 2013; Li‐Harms *et al*. 2015; Diot *et al*. 2016). Interestingly, these mice presented an increase in mitochondrial biogenesis regulator peroxisome proliferator‐activated receptor gamma coactivator‐1α (PGC‐1α) and its target proteins, mitochondrial transcription factor A (TFAM) and nuclear respiratory factor 1 in the muscle (Joseph *et al*. 2013), which might be a compensatory mechanism to counteract the increase in mitophagy.

Indeed, mitochondrial biogenesis is the process by which cells increase their individual mitochondrial mass in order to increase their energy production. This process is of utmost importance for cells like neurons having high requirement, and it is again not surprising that defects in biogenesis lead to neuronal impairments and neurodegeneration (Onyango *et al*. 2010). Evidence of an age‐related decrease in mitochondrial biogenesis has previously been reported, but the precise reason for this decrease remains elusive (Lopez‐Lluch *et al*. 2008; Onyango *et al*. 2010; Wenz 2011). As mentioned above, PGC‐1α plays a central role in biogenesis, especially in the brain where it participates to the maintenance of normal neuronal function as well as to the regulation of oxidative stress response (Wenz 2011). PGC‐1α was involved in the pathogenesis of neurodegenerative diseases, such as Huntington's or Parkinson's disease, but, to our knowledge, clear evidence of the regulation of this protein in the context of normal brain aging is still missing. Nevertheless, in a study comparing mitochondrial biogenesis in the frontal cortex from rats, authors showed a 25% loss in mtDNA content in aged (26‐month‐old) when compared to young (6‐month‐ old) animals, paralleled by a 35% increase in the mtDNA deletion content (Picca *et al*. 2013). Surprisingly, the level of TFAM, a target of PGC‐1α, presented an increase in 70% in the cortex of old rats, which did not correlate with the decrease in mtDNA content. However, a strong decrease in TFAM‐bound mtDNA (60–70%) was measured in aged animals that may be because of the mtDNA deletion observed with aging. These data indicate that impairments in mitochondrial biogenesis during aging may, at least in part, be as a result of the age‐related increase in mtDNA deletion leading to a decreased ability to replicate mtDNA and to generate new mitochondria. Further investigation is required to test this hypothesis and to unravel the underlying mechanisms.

Taken together, these findings suggest that mitochondrial dynamics, mitophagy and biogenesis are impaired with increasing age. Since these deficits were involved in the pathogenesis of neurodegenerative disorders, including Alzheimer's, Parkinson's and Huntington's disease (Itoh *et al*. 2013), deeper investigations need to be done in the brain in order to dissect into more details the molecular machinery leading either to ‘normal’ healthy aging, or, on the contrary, to pathological aging.

## Sex differences in age‐induced mitochondrial dysfunction

Since epidemiological studies have revealed sex differences in several neural pathologies, taking into account this parameter is becoming of great importance in the study of brain physiology, especially regarding age‐related mitochondrial dysfunction. Indeed, sexual dimorphism can be observed at different levels, from neuronal circuits to the concentration of neuroactive steroids in the central nervous system [reviewed in (Panzica and Melcangi 2016)]. Besides, increasing evidence showed that age‐dependent changes in brain bioenergetics and redox homeostasis are also subjected to sex differences (Yin *et al*. 2015; Grimm *et al*. 2016c; Zhao *et al*. 2016).

At the gene level, a study performed on 55 cognitively intact individuals (aged 20–99 years) revealed predominant age‐related changes in the male brain, in particular a down‐regulation of genes involved in ETC, OXPHOS, ribonucleotide metabolism, ATP metabolism/biosynthesis, and mitochondrial transport (Berchtold *et al*. 2008). This suggested that males present a decreased capacity for energy production with increasing age. Similar data were obtained when comparing young/old and female/male individuals, where age and/or sex related differences were detected in the expression of genes involved in mitochondrial function, including mitochondrial dynamics, and mitophagy (Guebel and Torres 2016).

Regarding the redox homeostasis, *in vivo* monitoring of GSH content in the human brain provided evidence of higher levels in young females (± 26 years old) in the frontal and parietal cortex compared to young men (Mandal *et al*. 2012), with a gradual decrease with aging, as observed in the brain of older females (± 56 years old). Of note, women appear to be better armed to fight against oxidative stress before menopause by having higher antioxidant defenses compared to men [reviewed in (Vina and Borras 2010)].

These features can also be observed in animals. Indeed, brain mitochondria from young female rats (4–6 months) exhibited lower peroxide production compared with males at the same age (Borras *et al*. 2003). Interestingly, after ovariectomy, mitochondrial peroxide levels were similar in males and females. This phenomenon was reversed by a treatment with estradiol, the most abundant estrogen in females, highlighting a protective effect of this steroid against oxidative stress. In line with these data, increased levels of lipid peroxidation, coupled with a decrease in the mitochondrial respiratory control ratio (RCR), pyruvate dehydrogenase as well as cytochrome c oxidase (COX) activity, were observed in the brain of 6‐month‐ old ovariectomy mice compared to sham operated animals (Yao *et al*. 2012). Again, a treatment with estradiol was able to prevent these disturbances in the brain energy metabolism. Of note, estradiol was not only efficient in females in reducing brain ROS production but also in gonadectomized males by increasing MnSOD activity (Razmara *et al*. 2007). Interestingly, Guevara and co‐workers showed that female rats were less affected by the age‐related changes in brain mitochondrial oxidative status compared with males, in part because of higher antioxidant capacity (GPx activity) and respiration (COX and ATPase activities) (Guevara *et al*. 2009, 2011a,b). However, in SAMR1 mice, sex differences in brain oxidative stress are visible only in young animals (5 months old) and disappeared with aging (in 10 months old male and females) (Escames *et al*. 2013). These findings suggest that young females are protected against oxidative stress and bioenergetic deficits, and that this protection is lost during aging.

Interestingly, in a study investigating the effects of aging on mitochondrial function in the brain of female mice, Roberta Brinton group showed that reproductive senescence was paralleled by a significant decline in mitochondrial RCR, especially between 9 and 12 months of age (Yao *et al*. 2010). Within the same time window, pyruvate dehydrogenase and COX activity were significantly decreased while oxidative stress increased. Further investigation of female mouse brains revealed an activation of the fatty acid metabolism machinery during reproductive senescence, coupled with a rise of brain ketone bodies as well as myelin degeneration (Yao *et al*. 2010; Klosinski *et al*. 2015). The authors suggested that the catabolism of myelin lipids to generate ketone bodies during female brain aging may constitute an adaptive system to sustain energy demand in response to mitochondrial dysfunction. A better understanding of the underlying mechanisms would help identify an optimal window of opportunity to prevent or delay age‐related bioenergetic deficits in females that may initiate neurodegenerative diseases, such as AD.

Since female reproductive aging is marked by a drop in the circulating levels of estradiol, one can suggest that this sex hormone exerts a protective effect, especially on mitochondrial function (reviewed in (Grimm *et al*. 2012, 2016c). Indeed, estradiol was shown to: (i) increase glucose metabolism by regulating the expression of genes involved in glucose transport, glycolysis and tricarboxylic acid cycle, (ii) up‐regulate genes encoding for components of the mitochondrial ETC. including complex I, cytochrome c oxidase (complex IV), and the F1 subunit of ATP synthase, (iii) up‐regulate anti‐oxidant defenses, such as MnSOD and GSH levels, (iv) modulate the redox state of cells by acting on several signaling pathways, such as mitogen activated protein kinase, G protein regulated signaling, NFκB, c‐fos, CREB (cAMP response element‐binding protein), phosphatidylinositol‐3‐kinase, PKC (protein kinase C) and Ca^2+^ influx. On the basis of this complex mode of action, estradiol is able to decrease oxidative stress markers, including lipid peroxidation, protein oxidation and DNA damage, and can also regulate mitochondrial respiratory function. These effects of estrogen are mainly mediated by nuclear estrogen receptors (ERα and β), stimulating the expression of genes by binding to the estrogen response elements in the nucleus (Klinge *et al*. 2004). It is very interesting to note that ERs are not only found in the nucleus but also within mitochondria where they modulate the expression of genes coded by the mtDNA. Mitochondrial ERα and β were found in different brain regions in female rats, namely the cortex, hippocampus and hypothalamus, and no differences were detected between young (3 months) and aged (18 months) animals (Alvarez‐Delgado *et al*. 2010). Thus, given the regulatory role that plays estradiol in mitochondrial function, the age‐related loss of this protective sex steroid (namely after the menopause in human) may constitute a risk factor leading to mitochondrial impairments and to the genesis of neurodegenerative disorders (Grimm *et al*. 2012, 2016c; Velarde 2014).

Estradiol may not be the only steroid involved in neuroprotection. Gaignard and collaborators also showed that the levels of other steroids, pregnenolone and progesterone, are higher in the brain of young females (3 months old) compared to age‐matched males and decreased with aging (Gaignard *et al*. 2015). This may also contribute to the sex differences observed in brain mitochondrial function. Of note, steroids can also be synthesized locally within the nervous system (independently of peripheral steroidogenic glands) where they are called ‘neurosteroids’ (Corpechot *et al*. 1981). Alteration in brain steroid levels were observed during aging (Caruso *et al*. 2013), and sex differences in the level of these neuroactive steroids have been documented [reviewed in (Melcangi *et al*. 2015)]. To gain insights into the effects of neurosteroids on mitochondria function, we recently showed that estrogens (estradiol and estrone), androgens (testosterone and 3α‐androstanediol), as well as dehydroepiandrosterone and allopregnanolone, are able to improve bioenergetics and antioxidant defenses *in vitro* (in SH‐SY5Y neuroblastoma cells and in primary neuronal cultures) by increasing ATP levels, mitochondrial membrane potential, basal respiration, and MnSOD activity (Grimm *et al*. 2014). Besides, a treatment with sex hormone‐related neurosteroids (progesterone, estrogens and androgens) as well as allopregnanolone was efficient to reduce bioenergetic impairments observed in cellular models of AD, indicating that neurosteroids represent attractive tools to counteract mitochondrial deficits in this neuropathology (Grimm *et al*. 2016a; Lejri *et al*. 2016).

Thus, sexual dimorphism can be observed in brain mitochondria that may be explained by the difference in sex hormones between male and females. Given the importance of sex in the development of neurodegenerative diseases (Rettberg *et al*. 2016; Zhao *et al*. 2016; Pike 2017), this important variable should be taken into account in further studies in order to understand the complex relationship between aging, mitochondria and sex hormones.

## Conclusions

**Figure 3:**
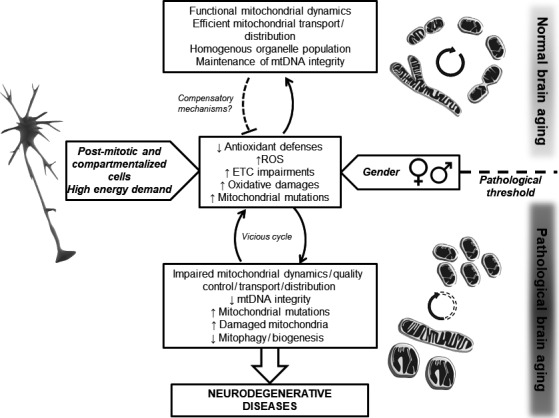
Model of the influence of mitochondrial function on brain aging. Increased oxidative stress is a characteristic of brain aging with an increase in reactive oxygen species (ROS) production and/or defects in the antioxidant system. Mitochondrial dynamics protect the cells (especially highly differentiated cells such as neurons) against the accumulation of mitochondrial mutations. When the system is in balance, this leads to normal aging. However, when a pathological threshold is passed (dashed line), impaired mitochondrial dynamics may lead to the accumulation of defective organelle, triggering a cascade of event inducing neurodegeneration. The fact that neurons are post‐mitotic compartmentalized cells, as well as gender differences, brings additional points of complexity to the system. ETC.; electron transport chain, mtDNA; mitochondrial DNA.

In this review, we aimed to look at brain aging processes from a mitochondrial point of view (Fig. 3), and we showed that:

- Mitochondria are at the center of the free radicals theory of aging by being a source and target of ROS. The age‐related increase in brain oxidative stress may lead to protein, lipid as well as DNA oxidation, which in turn affects mitochondrial function. When a pathological threshold is passed, this may trigger cell death by apoptosis (Fig. 1);
- Mitochondrial dynamics play an important role in maintaining a healthy organelle population (Fig. 2). Impairments in this quality control system may lead to the accumulation of defective mitochondria, as well as inefficient mitochondrial transport and distribution, again leading to synaptic and neuronal degeneration;
- Neurons are particularly vulnerable to oxidative insults and mitochondrial dysfunction given that they are post‐mitotic differentiated cells relying almost exclusively on the OXPHOS system to sustain their high energy needs. Besides, distinct mitochondrial populations can be observed in different neuronal compartments (e.g., synaptic vs. non‐synaptic), highlighting the importance of proper mitochondrial distribution in these highly compartmentalized cells;
- A sexual dimorphism (not systematically investigated in aging studies) can be observed in brain mitochondrial function and may explain sex differences in the physiopathology of neurodegenerative disorders

Of course, this picture is even more complex. ROS were also shown to extend longevity by acting as a signaling molecule, according to the concept of ‘mitohormesis’ , which states that mild mitochondrial stress would protect the cells against subsequent perturbations [reviewed in (Hekimi *et al*. 2016; Yun and Finkel 2014)]. Besides, mitochondria are not isolated semiautonomous organelles in the cells, but they are in physical contact with the endoplasmic reticulum via the mitochondrial‐associated ER membranes (Marchi *et al*. 2014). This interaction plays an important role in the regulation of intracellular calcium homeostasis, and appears to be also impaired in aging (Calvo‐Rodriguez *et al*. 2016). Finally, there is a growing interest in studying mitochondria‐nucleus signaling because: (i) on the one hand, mitochondria stress was shown to activate cytosolic signaling pathways regulating nuclear gene expression, and (ii) on the other hand, DNA damage signaling was shown to regulate mitophagy and apoptosis [reviewed in (Fang *et al*. 2016)].

Thus, further investigation needs to be done to unravel the underlying mechanism leading either to normal aging or on the contrary to neurodegenerative disease. A better understanding of mitochondrial physiology in the context of brain aging may help to identify therapeutic targets against neurodegeneration.

## References

[jnc14037-bib-0001] Alvarez‐Delgado C. , Mendoza‐Rodriguez C. A. , Picazo O. and Cerbon M. (2010) Different expression of alpha and beta mitochondrial estrogen receptors in the aging rat brain: interaction with respiratory complex V. Exp. Gerontol. 45, 580–585. 20096765 10.1016/j.exger.2010.01.015

[jnc14037-bib-0002] Amadoro G. , Corsetti V. , Florenzano F. , Atlante A. , Bobba A. , Nicolin V. , Nori S. L. and Calissano P. (2014) Morphological and bioenergetic demands underlying the mitophagy in post‐mitotic neurons: the pink‐parkin pathway. Front. Aging Neurosci. 6, 18.24600391

[jnc14037-bib-0003] Aon M. A. , Cortassa S. , Juhaszova M. and Sollott S. J. (2016) Mitochondrial health, the epigenome and healthspan. Clin. Sci. 130, 1285–1305. 27358026 10.1042/CS20160002 PMC5066813

[jnc14037-bib-0004] Barja G. (1999) Mitochondrial oxygen radical generation and leak: sites of production in states 4 and 3, organ specificity, and relation to aging and longevity. J. Bioenerg. Biomembr. 31, 347–366. 10665525 10.1023/a:1005427919188

[jnc14037-bib-0005] Batlevi Y. and La Spada A. R. (2011) Mitochondrial autophagy in neural function, neurodegenerative disease, neuron cell death, and aging. Neurobiol. Dis. 43, 46–51. 20887789 10.1016/j.nbd.2010.09.009 PMC3096708

[jnc14037-bib-0006] Berchtold N. C. , Cribbs D. H. , Coleman P. D. *et al* (2008) Gene expression changes in the course of normal brain aging are sexually dimorphic. Proc. Natl Acad. Sci. USA 105, 15605–15610. 18832152 10.1073/pnas.0806883105 PMC2563070

[jnc14037-bib-0007] Borras C. , Sastre J. , Garcia‐Sala D. , Lloret A. , Pallardo F. V. and Vina J. (2003) Mitochondria from females exhibit higher antioxidant gene expression and lower oxidative damage than males. Free Radic. Biol. Med. 34, 546–552. 12614843 10.1016/s0891-5849(02)01356-4

[jnc14037-bib-0008] Borras C. , Gambini J. , Lopez‐Grueso R. , Pallardo F. V. and Vina J. (2010) Direct antioxidant and protective effect of estradiol on isolated mitochondria. Biochem. Biophys. Acta. 1802, 205–211. 19751829 10.1016/j.bbadis.2009.09.007

[jnc14037-bib-0009] Brown G. C. and Borutaite V. (2002) Nitric oxide inhibition of mitochondrial respiration and its role in cell death. Free Radic. Biol. Med. 33, 1440–1450. 12446201 10.1016/s0891-5849(02)01112-7

[jnc14037-bib-0010] Calvo‐Rodriguez M. , Garcia‐Durillo M. , Villalobos C. and Nunez L. (2016) In vitro aging promotes endoplasmic reticulum (ER)‐mitochondria Ca2 + cross talk and loss of store‐operated Ca2 + entry (SOCE) in rat hippocampal neurons. Biochim. Biophys. Acta 1863, 2637–2649. 27503411 10.1016/j.bbamcr.2016.08.001

[jnc14037-bib-0011] Campello S. and Scorrano L. (2010) Mitochondrial shape changes: orchestrating cell pathophysiology. EMBO Rep. 11, 678–684. 20725092 10.1038/embor.2010.115 PMC2933866

[jnc14037-bib-0012] Carelli V. , Maresca A. , Caporali L. , Trifunov S. , Zanna C. and Rugolo M. (2015) Mitochondria: Biogenesis and mitophagy balance in segregation and clonal expansion of mitochondrial DNA mutations. Int. J. Biochem. Cell Biol. 63, 21–24. 25666555 10.1016/j.biocel.2015.01.023

[jnc14037-bib-0013] Caruso D. , Barron A. M. , Brown M. A. , Abbiati F. , Carrero P. , Pike C. J. , Garcia‐Segura L. M. and Melcangi R. C. (2013) Age‐related changes in neuroactive steroid levels in 3xTg‐AD mice. Neurobiol. Aging 34, 1080–1089. 23122920 10.1016/j.neurobiolaging.2012.10.007 PMC3545103

[jnc14037-bib-0014] Chan D. C. (2006) Mitochondria: dynamic organelles in disease, aging, and development. Cell 125, 1241–1252. 16814712 10.1016/j.cell.2006.06.010

[jnc14037-bib-0015] Chan D. C. (2012) Fusion and fission: interlinked processes critical for mitochondrial health. Annu. Rev. Genet. 46, 265–287. 22934639 10.1146/annurev-genet-110410-132529

[jnc14037-bib-0016] Chauhan A. , Vera J. and Wolkenhauer O. (2014) The systems biology of mitochondrial fission and fusion and implications for disease and aging. Biogerontology 15, 1–12. 24122214 10.1007/s10522-013-9474-z

[jnc14037-bib-0017] Chen H. , Vermulst M. , Wang Y. E. , Chomyn A. , Prolla T. A. , McCaffery J. M. and Chan D. C. (2010) Mitochondrial fusion is required for mtDNA stability in skeletal muscle and tolerance of mtDNA mutations. Cell 141, 280–289. 20403324 10.1016/j.cell.2010.02.026 PMC2876819

[jnc14037-bib-0018] Corpechot C. , Robel P. , Axelson M. , Sjovall J. and Baulieu E. E. (1981) Characterization and measurement of dehydroepiandrosterone sulfate in rat brain. Proc. Natl Acad. Sci. USA 78, 4704–4707. 6458035 10.1073/pnas.78.8.4704 PMC320231

[jnc14037-bib-0019] DeBalsi K. L. , Hoff K. E. and Copeland W. C. (2016) Role of the mitochondrial DNA replication machinery in mitochondrial DNA mutagenesis, aging and age‐related diseases. Ageing Res. Rev. 33, 89–104. 27143693 10.1016/j.arr.2016.04.006 PMC5086445

[jnc14037-bib-0020] Diot A. , Hinks‐Roberts A. , Lodge T. *et al* (2015) A novel quantitative assay of mitophagy: combining high content fluorescence microscopy and mitochondrial DNA load to quantify mitophagy and identify novel pharmacological tools against pathogenic heteroplasmic mtDNA. Pharmacol. Res. 100, 24–35. 26196248 10.1016/j.phrs.2015.07.014

[jnc14037-bib-0021] Diot A. , Morten K. and Poulton J. (2016) Mitophagy plays a central role in mitochondrial ageing. Mamm. Genome 27, 381–395. 27352213 10.1007/s00335-016-9651-x PMC4935730

[jnc14037-bib-0022] Escames G. , Diaz‐Casado M. E. , Doerrier C. , Luna‐Sanchez M. , Lopez L. C. and Acuna‐Castroviejo D. (2013) Early gender differences in the redox status of the brain mitochondria with age: effects of melatonin therapy. Horm. Mol. Biol. Clin. Investig 16, 91–100. 10.1515/hmbci-2013-0026 25436750

[jnc14037-bib-0023] Fang E. F. , Scheibye‐Knudsen M. , Chua K. F. , Mattson M. P. , Croteau D. L. and Bohr V. A. (2016) Nuclear DNA damage signalling to mitochondria in ageing. Nat. Rev. Mol. Cell Biol. 17, 308–321. 26956196 10.1038/nrm.2016.14 PMC5161407

[jnc14037-bib-0024] Friedman J. R. and Nunnari J. (2014) Mitochondrial form and function. Nature 505, 335–343. 24429632 10.1038/nature12985 PMC4075653

[jnc14037-bib-0025] Gaignard P. , Savouroux S. , Liere P. , Pianos A. , Therond P. , Schumacher M. , Slama A. and Guennoun R. (2015) Effect of sex differences on brain mitochondrial function and its suppression by ovariectomy and in aged mice. Endocrinology 156, 2893–2904. 26039154 10.1210/en.2014-1913

[jnc14037-bib-0026] Goldstein S. and Merenyi G. (2008) The chemistry of peroxynitrite: implications for biological activity. Methods Enzymol. 436, 49–61. 18237627 10.1016/S0076-6879(08)36004-2

[jnc14037-bib-0027] Grimm A. , Lim Y. A. , Mensah‐Nyagan A. G. , Götz J. and Eckert A. (2012) Alzheimer's disease, oestrogen and mitochondria: an ambiguous relationship. Mol. Neurobiol. 46, 151–160. 22678467 10.1007/s12035-012-8281-x PMC3443477

[jnc14037-bib-0028] Grimm A. , Schmitt K. , Lang U. E. , Mensah‐Nyagan A. G. and Eckert A. (2014) Improvement of neuronal bioenergetics by neurosteroids: implications for age‐related neurodegenerative disorders. Biochim. Biophys. Acta 1842, 2427–2438. 25281013 10.1016/j.bbadis.2014.09.013

[jnc14037-bib-0029] Grimm A. , Biliouris E. E. , Lang U. E. , Götz J. , Mensah‐Nyagan A. G. and Eckert A. (2016a) Sex hormone‐related neurosteroids differentially rescue bioenergetic deficits induced by amyloid‐beta or hyperphosphorylated tau protein. Cell. Mol. Life Sci. 73, 201–215. 26198711 10.1007/s00018-015-1988-x PMC4700074

[jnc14037-bib-0030] Grimm A. , Friedland K. and Eckert A. (2016b) Mitochondrial dysfunction: the missing link between aging and sporadic Alzheimer's disease. Biogerontology 17, 281–296. 26468143 10.1007/s10522-015-9618-4

[jnc14037-bib-0031] Grimm A. , Mensah‐Nyagan A. G. and Eckert A. (2016c) Alzheimer, mitochondria and gender. Neurosci. Biobehav. Rev. 67, 89–101. 27139022 10.1016/j.neubiorev.2016.04.012

[jnc14037-bib-0032] Guebel D. V. and Torres N. V. (2016) Sexual dimorphism and aging in the human hyppocampus: identification, validation, and impact of differentially expressed genes by factorial microarray and network analysis. Front. Aging Neurosci. 8, 229. 27761111 10.3389/fnagi.2016.00229 PMC5050216

[jnc14037-bib-0033] Guevara R. , Santandreu F. M. , Valle A. , Gianotti M. , Oliver J. and Roca P. (2009) Sex‐dependent differences in aged rat brain mitochondrial function and oxidative stress. Free Radic. Biol. Med. 46, 169–175. 18992805 10.1016/j.freeradbiomed.2008.09.035

[jnc14037-bib-0034] Guevara R. , Gianotti M. , Oliver J. and Roca P. (2011a) Age and sex‐related changes in rat brain mitochondrial oxidative status. Exp. Gerontol. 46, 923–928. 21864669 10.1016/j.exger.2011.08.003

[jnc14037-bib-0035] Guevara R. , Gianotti M. , Roca P. and Oliver J. (2011b) Age and sex‐related changes in rat brain mitochondrial function. Cell. Physiol. Biochem. 27, 201–206. 21471708 10.1159/000327945

[jnc14037-bib-0036] Harman D. (1956) Aging: a theory based on free radical and radiation chemistry. J. Gerontol. 11, 298–300. 13332224 10.1093/geronj/11.3.298

[jnc14037-bib-0037] Harman D. (1981) The aging process. Proc. Natl Acad. Sci. USA 78, 7124–7128. 6947277 10.1073/pnas.78.11.7124 PMC349208

[jnc14037-bib-0038] Haroon S. and Vermulst M. (2016) Linking mitochondrial dynamics to mitochondrial protein quality control. Curr. Opin. Genet. Dev. 38, 68–74. 27235806 10.1016/j.gde.2016.04.004

[jnc14037-bib-0039] Hauser D. N. , Dillman A. A. , Ding J. , Li Y. and Cookson M. R. (2014) Post‐translational decrease in respiratory chain proteins in the Polg mutator mouse brain. PLoS ONE 9, e94646. 24722488 10.1371/journal.pone.0094646 PMC3983222

[jnc14037-bib-0040] Hekimi S. , Wang Y. and Noe A. (2016) Mitochondrial ROS and the effectors of the intrinsic apoptotic pathway in aging cells: the discerning killers!. Front. Genet. 7, 161. 27683586 10.3389/fgene.2016.00161 PMC5021979

[jnc14037-bib-0041] Itoh K. , Nakamura K. , Iijima M. and Sesaki H. (2013) Mitochondrial dynamics in neurodegeneration. Trends Cell Biol. 23, 64–71. 23159640 10.1016/j.tcb.2012.10.006 PMC3558617

[jnc14037-bib-0042] Jezek P. and Hlavata L. (2005) Mitochondria in homeostasis of reactive oxygen species in cell, tissues, and organism. Inter. J. Biochem. Cell Biol. 37, 2478–2503. 10.1016/j.biocel.2005.05.013 16103002

[jnc14037-bib-0043] Joseph A. M. , Adhihetty P. J. , Wawrzyniak N. R. , Wohlgemuth S. E. , Picca A. , Kujoth G. C. , Prolla T. A. and Leeuwenburgh C. (2013) Dysregulation of mitochondrial quality control processes contribute to sarcopenia in a mouse model of premature aging. PLoS ONE 8, e69327. 23935986 10.1371/journal.pone.0069327 PMC3720551

[jnc14037-bib-0044] Kageyama Y. , Zhang Z. , Roda R. *et al* (2012) Mitochondrial division ensures the survival of postmitotic neurons by suppressing oxidative damage. J. Cell Biol. 197, 535–551. 22564413 10.1083/jcb.201110034 PMC3352955

[jnc14037-bib-0045] Kauppila T. E. S. , Kauppila J. H. K. and Larsson N. G. (2016) Mammalian Mitochondria and Aging: an Update. Cell Metab. 25, 57–71. 28094012 10.1016/j.cmet.2016.09.017

[jnc14037-bib-0046] Keil U. , Bonert A. , Marques C. A. *et al* (2004) Amyloid beta‐induced changes in nitric oxide production and mitochondrial activity lead to apoptosis. J. Biol. Chem. 279, 50310–50320. 15371443 10.1074/jbc.M405600200

[jnc14037-bib-0047] Kishida K. T. and Klann E. (2007) Sources and targets of reactive oxygen species in synaptic plasticity and memory. Antioxid. Redox Signal. 9, 233–244. 17115936 10.1089/ars.2007.9.ft-8 PMC3544198

[jnc14037-bib-0048] Klinge C. M. , Jernigan S. C. , Mattingly K. A. , Risinger K. E. and Zhang J. (2004) Estrogen response element‐dependent regulation of transcriptional activation of estrogen receptors alpha and beta by coactivators and corepressors. J. Mol. Endocrinol. 33, 387–410. 15525597 10.1677/jme.1.01541

[jnc14037-bib-0049] Klosinski L. P. , Yao J. , Yin F. , Fonteh A. N. , Harrington M. G. , Christensen T. A. , Trushina E. and Brinton R. D. (2015) White matter lipids as a ketogenic fuel supply in aging female brain: implications for Alzheimer's disease. EBioMedicine 2, 1888–1904. 26844268 10.1016/j.ebiom.2015.11.002 PMC4703712

[jnc14037-bib-0050] Knott A. B. , Perkins G. , Schwarzenbacher R. and Bossy‐Wetzel E. (2008) Mitochondrial fragmentation in neurodegeneration. Nat. Rev. Neurosci. 9, 505–518. 18568013 10.1038/nrn2417 PMC2711514

[jnc14037-bib-0051] Kowald A. and Kirkwood T. B. (2000) Accumulation of defective mitochondria through delayed degradation of damaged organelles and its possible role in the ageing of post‐mitotic and dividing cells. J. Theor. Biol. 202, 145–160. 10640434 10.1006/jtbi.1999.1046

[jnc14037-bib-0052] Krestinina O. , Azarashvili T. , Baburina Y. , Galvita A. , Grachev D. , Stricker R. and Reiser G. (2015) In aging, the vulnerability of rat brain mitochondria is enhanced due to reduced level of 2’,3’‐cyclic nucleotide‐3’‐phosphodiesterase (CNP) and subsequently increased permeability transition in brain mitochondria in old animals. Neurochem. Int. 80, 41–50. 25277077 10.1016/j.neuint.2014.09.008

[jnc14037-bib-0053] Kujoth G. C. , Hiona A. , Pugh T. D. *et al* (2005) Mitochondrial DNA mutations, oxidative stress, and apoptosis in mammalian aging. Science 309, 481–484. 16020738 10.1126/science.1112125

[jnc14037-bib-0054] Kurokawa T. , Asada S. , Nishitani S. and Hazeki O. (2001) Age‐related changes in manganese superoxide dismutase activity in the cerebral cortex of senescence‐accelerated prone and resistant mouse. Neurosci. Lett. 298, 135–138. 11163296 10.1016/s0304-3940(00)01755-9

[jnc14037-bib-0055] Lejri I. , Grimm A. , Miesch M. , Geoffroy P. , Eckert A. and Mensah‐Nyagan A. G. (2016) Allopregnanolone and its analog BR 297 rescue neuronal cells from oxidative stress‐induced death through bioenergetic improvement. Biochim. Biophys. Acta 1863, 631–642. 27979708 10.1016/j.bbadis.2016.12.007

[jnc14037-bib-0056] Leuner K. , Hauptmann S. , Abdel‐Kader R. , Scherping I. , Keil U. , Strosznajder J. B. , Eckert A. and Muller W. E. (2007) Mitochondrial dysfunction: the first domino in brain aging and Alzheimer's disease? Antioxid. Redox Signal. 9, 1659–1675. 17867931 10.1089/ars.2007.1763

[jnc14037-bib-0057] Leuner K. , Schutt T. , Kurz C. *et al* (2012) Mitochondrion‐derived reactive oxygen species lead to enhanced amyloid beta formation. Antioxid. Redox Signal. 16, 1421–1433. 22229260 10.1089/ars.2011.4173 PMC3329950

[jnc14037-bib-0058] Li‐Harms X. , Milasta S. , Lynch J. *et al* (2015) Mito‐protective autophagy is impaired in erythroid cells of aged mtDNA‐mutator mice. Blood 125, 162–174. 25411424 10.1182/blood-2014-07-586396 PMC4281825

[jnc14037-bib-0059] Lin M. Y. and Sheng Z. H. (2015) Regulation of mitochondrial transport in neurons. Exp. Cell Res. 334, 35–44. 25612908 10.1016/j.yexcr.2015.01.004 PMC4433773

[jnc14037-bib-0060] Lopez‐Lluch G. , Irusta P. M. , Navas P. and de Cabo R. (2008) Mitochondrial biogenesis and healthy aging. Exp. Gerontol. 43, 813–819. 18662766 10.1016/j.exger.2008.06.014 PMC2562606

[jnc14037-bib-0061] Lores‐Arnaiz S. and Bustamante J. (2011) Age‐related alterations in mitochondrial physiological parameters and nitric oxide production in synaptic and non‐synaptic brain cortex mitochondria. Neuroscience 188, 117–124. 21600964 10.1016/j.neuroscience.2011.04.060

[jnc14037-bib-0062] Lores‐Arnaiz S. , Lombardi P. , Karadayian A. G. , Orgambide F. , Cicerchia D. and Bustamante J. (2016) Brain cortex mitochondrial bioenergetics in synaptosomes and non‐synaptic mitochondria during aging. Neurochem. Res. 41, 353–363. 26818758 10.1007/s11064-015-1817-5

[jnc14037-bib-0063] Mandal P. K. , Tripathi M. and Sugunan S. (2012) Brain oxidative stress: detection and mapping of anti‐oxidant marker ‘Glutathione’ in different brain regions of healthy male/female, MCI and Alzheimer patients using non‐invasive magnetic resonance spectroscopy. Biochem. Biophys. Res. Commun. 417, 43–48. 22120629 10.1016/j.bbrc.2011.11.047

[jnc14037-bib-0064] Marchi S. , Patergnani S. and Pinton P. (2014) The endoplasmic reticulum‐mitochondria connection: one touch, multiple functions. Biochim. Biophys. Acta 1837, 461–469. 24211533 10.1016/j.bbabio.2013.10.015

[jnc14037-bib-0065] Mattson M. P. , Gleichmann M. and Cheng A. (2008) Mitochondria in neuroplasticity and neurological disorders. Neuron 60, 748–766. 19081372 10.1016/j.neuron.2008.10.010 PMC2692277

[jnc14037-bib-0066] Melcangi R. C. , Giatti S. and Garcia‐Segura L. M. (2015) Levels and actions of neuroactive steroids in the nervous system under physiological and pathological conditions: sex‐specific features. Neurosci. Biobehav. Rev. 67, 25–40. 26657814 10.1016/j.neubiorev.2015.09.023

[jnc14037-bib-0067] Nakahara H. , Kanno T. , Inai Y. , Utsumi K. , Hiramatsu M. , Mori A. and Packer L. (1998) Mitochondrial dysfunction in the senescence accelerated mouse (SAM). Free Radic. Biol. Med. 24, 85–92. 9436617 10.1016/s0891-5849(97)00164-0

[jnc14037-bib-0068] Navarro A. and Boveris A. (2004) Rat brain and liver mitochondria develop oxidative stress and lose enzymatic activities on aging. Am. J. Physiol. Regul. Integr. Comp. Physiol. 287, R1244–R1249. 15271654 10.1152/ajpregu.00226.2004

[jnc14037-bib-0069] Niccoli T. and Partridge L. (2012) Ageing as a risk factor for disease. Curr. Biol. 22, R741–R752. 22975005 10.1016/j.cub.2012.07.024

[jnc14037-bib-0070] Nunnari J. and Suomalainen A. (2012) Mitochondria: in sickness and in health. Cell 148, 1145–1159. 22424226 10.1016/j.cell.2012.02.035 PMC5381524

[jnc14037-bib-0071] Obashi K. and Okabe S. (2013) Regulation of mitochondrial dynamics and distribution by synapse position and neuronal activity in the axon. Eur. J. Neurosci. 38, 2350–2363. 23725294 10.1111/ejn.12263

[jnc14037-bib-0072] Oettinghaus B. , Schulz J. M. , Restelli L. M. *et al* (2016) Synaptic dysfunction, memory deficits and hippocampal atrophy due to ablation of mitochondrial fission in adult forebrain neurons. Cell Death Differ. 23, 18–28. 25909888 10.1038/cdd.2015.39 PMC4815974

[jnc14037-bib-0073] Omata N. , Murata T. , Fujibayashi Y. , Waki A. , Sadato N. , Yoshimoto M. , Wada Y. and Yonekura Y. (2001) Age‐related changes in energy production in fresh senescence‐accelerated mouse brain slices as revealed by positron autoradiography. Dement. Geriatr. Cogn. Disord. 12, 78–84. 11173878 10.1159/000051239

[jnc14037-bib-0074] Onyango I. G. , Lu J. , Rodova M. , Lezi E. , Crafter A. B. and Swerdlow R. H. (2010) Regulation of neuron mitochondrial biogenesis and relevance to brain health. Biochim. Biophys. Acta 1802, 228–234. 19682571 10.1016/j.bbadis.2009.07.014

[jnc14037-bib-0075] Pandya J. D. , Grondin R. , Yonutas H. M. , Haghnazar H. , Gash D. M. , Zhang Z. and Sullivan P. G. (2015) Decreased mitochondrial bioenergetics and calcium buffering capacity in the basal ganglia correlates with motor deficits in a nonhuman primate model of aging. Neurobiol. Aging 36, 1903–1913. 25726361 10.1016/j.neurobiolaging.2015.01.018

[jnc14037-bib-0076] Panzica G. and Melcangi R. C. (2016) Structural and molecular brain sexual differences: a tool to understand sex differences in health and disease. Neurosci. Biobehav. Rev. 67, 2–8. 27113294 10.1016/j.neubiorev.2016.04.017

[jnc14037-bib-0077] Paradies G. , Petrosillo G. , Paradies V. and Ruggiero F. M. (2010) Oxidative stress, mitochondrial bioenergetics, and cardiolipin in aging. Free Radic. Biol. Med. 48, 1286–1295. 20176101 10.1016/j.freeradbiomed.2010.02.020

[jnc14037-bib-0078] Pernas L. and Scorrano L. (2016) Mito‐morphosis: mitochondrial fusion, fission, and cristae remodeling as key mediators of cellular function. Annu. Rev. Physiol. 78, 505–531. 26667075 10.1146/annurev-physiol-021115-105011

[jnc14037-bib-0079] Peskind E. R. , Li G. , Shofer J. B. *et al* (2014) Influence of lifestyle modifications on age‐related free radical injury to brain. JAMA Neurol. 71, 1150–1154. 25048271 10.1001/jamaneurol.2014.1428 PMC4160350

[jnc14037-bib-0080] Picca A. , Fracasso F. , Pesce V. , Cantatore P. , Joseph A. M. , Leeuwenburgh C. , Gadaleta M. N. and Lezza A. M. (2013) Age‐ and calorie restriction‐related changes in rat brain mitochondrial DNA and TFAM binding. Age 35, 1607–1620. 22945739 10.1007/s11357-012-9465-z PMC3776104

[jnc14037-bib-0081] Picca A. , Pesce V. , Sirago G. , Fracasso F. , Leeuwenburgh C. and Lezza A. M. (2016) “What makes some rats live so long?” The mitochondrial contribution to longevity through balance of mitochondrial dynamics and mtDNA content. Exp. Gerontol. 85, 33–40. 27620821 10.1016/j.exger.2016.09.010 PMC5922457

[jnc14037-bib-0082] Pike C. J. (2017) Sex and the development of Alzheimer's disease. J. Neurosci. Res. 95, 671–680. 27870425 10.1002/jnr.23827 PMC5120614

[jnc14037-bib-0083] Prince M. , Bryce R. , Albanese E. , Wimo A. , Ribeiro W. and Ferri C. P. (2013) The global prevalence of dementia: a systematic review and metaanalysis. Alzheimer's Dement 9, e62. 10.1016/j.jalz.2012.11.007 23305823

[jnc14037-bib-0084] Rana A. , Rera M. and Walker D. W. (2013) Parkin overexpression during aging reduces proteotoxicity, alters mitochondrial dynamics, and extends lifespan. Proc. Natl Acad. Sci. USA 110, 8638–8643. 23650379 10.1073/pnas.1216197110 PMC3666724

[jnc14037-bib-0085] Razmara A. , Duckles S. P. , Krause D. N. and Procaccio V. (2007) Estrogen suppresses brain mitochondrial oxidative stress in female and male rats. Brain Res. 1176, 71–81. 17889838 10.1016/j.brainres.2007.08.036 PMC2099309

[jnc14037-bib-0086] Rebrin I. , Forster M. J. and Sohal R. S. (2007) Effects of age and caloric intake on glutathione redox state in different brain regions of C57BL/6 and DBA/2 mice. Brain Res. 1127, 10–18. 17113050 10.1016/j.brainres.2006.10.040 PMC2112744

[jnc14037-bib-0087] Reifschneider N. H. , Goto S. , Nakamoto H. , Takahashi R. , Sugawa M. , Dencher N. A. and Krause F. (2006) Defining the mitochondrial proteomes from five rat organs in a physiologically significant context using 2D blue‐native/SDS‐PAGE. J. Proteome Res. 5, 1117–1132. 16674101 10.1021/pr0504440

[jnc14037-bib-0088] Rettberg J. R. , Dang H. , Hodis H. N. , Henderson V. W. , St John J. A. , Mack W. J. and Brinton R. D. (2016) Identifying postmenopausal women at risk for cognitive decline within a healthy cohort using a panel of clinical metabolic indicators: potential for detecting an at‐Alzheimer's risk metabolic phenotype. Neurobiol. Aging 40, 155–163. 26973115 10.1016/j.neurobiolaging.2016.01.011 PMC4921204

[jnc14037-bib-0089] Rolfe D. F. , Hulbert A. J. and Brand M. D. (1994) Characteristics of mitochondrial proton leak and control of oxidative phosphorylation in the major oxygen‐consuming tissues of the rat. Biochim. Biophys. Acta 1188, 405–416. 7803454 10.1016/0005-2728(94)90062-0

[jnc14037-bib-0090] Santos R. X. , Correia S. C. , Zhu X. , Smith M. A. , Moreira P. I. , Castellani R. J. , Nunomura A. and Perry G. (2013) Mitochondrial DNA oxidative damage and repair in aging and Alzheimer's disease. Antioxid. Redox Signal. 18, 2444–2457. 23216311 10.1089/ars.2012.5039 PMC3671662

[jnc14037-bib-0091] Sastre J. , Millan A. , Garcia de la Asuncion J. *et al* (1998) A Ginkgo biloba extract (EGb 761) prevents mitochondrial aging by protecting against oxidative stress. Free Radic. Biol. Med. 24, 298–304. 9433905 10.1016/s0891-5849(97)00228-1

[jnc14037-bib-0092] Scheibye‐Knudsen M. (2016) Neurodegeneration in accelerated aging. Danish Med. J., 63, 1–21. 27808039

[jnc14037-bib-0093] Schonfeld P. and Reiser G. (2013) Why does brain metabolism not favor burning of fatty acids to provide energy? Reflections on disadvantages of the use of free fatty acids as fuel for brain. J. Cereb. Blood Flow Metab. 33, 1493–1499. 23921897 10.1038/jcbfm.2013.128 PMC3790936

[jnc14037-bib-0094] Shi C. , Xiao S. , Liu J. , Guo K. , Wu F. , Yew D. T. and Xu J. (2010) Ginkgo biloba extract EGb761 protects against aging‐associated mitochondrial dysfunction in platelets and hippocampi of SAMP8 mice. Platelets 21, 373–379. 20459350 10.3109/09537100903511448

[jnc14037-bib-0095] Stauch K. L. , Purnell P. R. and Fox H. S. (2014) Aging synaptic mitochondria exhibit dynamic proteomic changes while maintaining bioenergetic function. Aging 6, 320–334. 24827396 10.18632/aging.100657 PMC4032798

[jnc14037-bib-0096] Terman A. , Kurz T. , Navratil M. , Arriaga E. A. and Brunk U. T. (2010) Mitochondrial turnover and aging of long‐lived postmitotic cells: the mitochondrial‐lysosomal axis theory of aging. Antioxid. Redox Signal. 12, 503–535. 19650712 10.1089/ars.2009.2598 PMC2861545

[jnc14037-bib-0097] Todorova V. and Blokland A. (2016) Mitochondria and synaptic plasticity in the mature and aging nervous system. Curr. Neuropharmacol. 15, 166–173. 10.2174/1570159X14666160414111821 PMC5327446 27075203

[jnc14037-bib-0098] Toman J. and Fiskum G. (2011) Influence of aging on membrane permeability transition in brain mitochondria. J. Bioenerg. Biomembr. 43, 3–10. 21311961 10.1007/s10863-011-9337-8 PMC4085790

[jnc14037-bib-0099] Trifunovic A. , Wredenberg A. , Falkenberg M. *et al* (2004) Premature ageing in mice expressing defective mitochondrial DNA polymerase. Nature 429, 417–423. 15164064 10.1038/nature02517

[jnc14037-bib-0100] Trifunovic A. , Hansson A. , Wredenberg A. *et al* (2005) Somatic mtDNA mutations cause aging phenotypes without affecting reactive oxygen species production. Proc. Natl Acad. Sci. USA 102, 17993–17998. 16332961 10.1073/pnas.0508886102 PMC1312403

[jnc14037-bib-0101] Twig G. and Shirihai O. S. (2011) The interplay between mitochondrial dynamics and mitophagy. Antioxid. Redox Signal. 14, 1939–1951. 21128700 10.1089/ars.2010.3779 PMC3078508

[jnc14037-bib-0102] Twig G. , Elorza A. , Molina A. J. *et al* (2008) Fission and selective fusion govern mitochondrial segregation and elimination by autophagy. EMBO J. 27, 433–446. 18200046 10.1038/sj.emboj.7601963 PMC2234339

[jnc14037-bib-0103] Veal E. A. , Day A. M. and Morgan B. A. (2007) Hydrogen peroxide sensing and signaling. Mol. Cell 26, 1–14. 17434122 10.1016/j.molcel.2007.03.016

[jnc14037-bib-0104] Velarde M. C. (2014) Mitochondrial and sex steroid hormone crosstalk during aging. Longev Healthspan 3, 2. 24495597 10.1186/2046-2395-3-2 PMC3922316

[jnc14037-bib-0105] Venkateshappa C. , Harish G. , Mahadevan A. , Srinivas Bharath M. M. and Shankar S. K. (2012) Elevated oxidative stress and decreased antioxidant function in the human hippocampus and frontal cortex with increasing age: implications for neurodegeneration in Alzheimer's disease. Neurochem. Res. 37, 1601–1614. 22461064 10.1007/s11064-012-0755-8

[jnc14037-bib-0106] Vina J. and Borras C. (2010) Women live longer than men: understanding molecular mechanisms offers opportunities to intervene by using estrogenic compounds. Antioxid. Redox Signal. 13, 269–278. 20059401 10.1089/ars.2009.2952

[jnc14037-bib-0107] Wang J. , Lei H. , Hou J. and Liu J. (2015) Involvement of oxidative stress in SAMP10 mice with age‐related neurodegeneration. Neurol. Sci. 36, 743–750. 25491662 10.1007/s10072-014-2029-5

[jnc14037-bib-0108] Wenz T. (2011) Mitochondria and PGC‐1alpha in Aging and Age‐Associated Diseases. J. Aging Res. 2011, 810619. 21629705 10.4061/2011/810619 PMC3100651

[jnc14037-bib-0109] Xu J. , Shi C. , Li Q. , Wu J. , Forster E. L. and Yew D. T. (2007) Mitochondrial dysfunction in platelets and hippocampi of senescence‐accelerated mice. J. Bioenerg. Biomembr. 39, 195–202. 17436064 10.1007/s10863-007-9077-y

[jnc14037-bib-0110] Yao J. , Hamilton R. T. , Cadenas E. and Brinton R. D. (2010) Decline in mitochondrial bioenergetics and shift to ketogenic profile in brain during reproductive senescence. Biochim. Biophys. Acta 1800, 1121–1126. 20538040 10.1016/j.bbagen.2010.06.002 PMC3200365

[jnc14037-bib-0111] Yao J. , Irwin R. , Chen S. , Hamilton R. , Cadenas E. and Brinton R. D. (2012) Ovarian hormone loss induces bioenergetic deficits and mitochondrial beta‐amyloid. Neurobiol. Aging 33, 1507–1521. 21514693 10.1016/j.neurobiolaging.2011.03.001 PMC3181273

[jnc14037-bib-0112] Yin F. , Yao J. , Sancheti H. *et al* (2015) The perimenopausal aging transition in the female rat brain: decline in bioenergetic systems and synaptic plasticity. Neurobiol. Aging 36, 2282–2295. 25921624 10.1016/j.neurobiolaging.2015.03.013 PMC4416218

[jnc14037-bib-0113] Youle R. J. and Narendra D. P. (2011) Mechanisms of mitophagy. Nat. Rev. Mol. Cell Biol. 12, 9–14. 21179058 10.1038/nrm3028 PMC4780047

[jnc14037-bib-0114] Yun J. and Finkel T. (2014) Mitohormesis. Cell Metab. 19, 757–766. 24561260 10.1016/j.cmet.2014.01.011 PMC4016106

[jnc14037-bib-0115] Zhang D. , Mott J. L. , Chang S. W. , Denniger G. , Feng Z. and Zassenhaus H. P. (2000) Construction of transgenic mice with tissue‐specific acceleration of mitochondrial DNA mutagenesis. Genomics 69, 151–161. 11031098 10.1006/geno.2000.6333

[jnc14037-bib-0116] Zhao L. , Mao Z. , Woody S. K. and Brinton R. D. (2016) Sex differences in metabolic aging of the brain: insights into female susceptibility to Alzheimer's disease. Neurobiol. Aging 42, 69–79. 27143423 10.1016/j.neurobiolaging.2016.02.011 PMC5644989

